# Open-Source Python Module for the Analysis of Personalized Light Exposure Data from Wearable Light Loggers and Dosimeters

**DOI:** 10.1080/15502724.2023.2296863

**Published:** 2024-02-28

**Authors:** Grégory Hammad, Katharina Wulff, Debra J. Skene, Mirjam Münch, Manuel Spitschan

**Affiliations:** a Sleep & Chronobiology Group, GIGA – CRC in Vivo Imaging, University of Liège, Liège, Belgium; b Chair of Neurogenetics, Institute of Human Genetics, University Hospital, Technical University of Munich, Munich, Germany; c Department of Molecular Biology, Umea University, Umea, Sweden; d Wallenberg Centre for Molecular Medicine (WCMM), Umea University, Umea, Sweden; e Chronobiology, Faculty of Health and Medical Sciences, University of Surrey, Guildford, UK; f Centre for Chronobiology, Psychiatric Hospital of the University of Basel, Basel, Switzerland; g Transfaculty Platform for Molecular and Cognitive Neuroscience, University of Basel, Basel, Switzerland; h Translational Sensory & Circadian Neuroscience, Max Planck Institute for Biological Cybernetics, Tübingen, Germany; i TUM School of Medicine & Health, Technical University of Munich, Munich, Germany; j TUM Institute for Advanced Study, Technical University of Munich, Garching, Germany

**Keywords:** Light exposure, dosimetry, light loggers, python, open-source software, analysis

## Abstract

Light exposure fundamentally influences human physiology and behavior, with light being the most important *zeitgeber* of the circadian system. Throughout the day, people are exposed to various scenes differing in light level, spectral composition and spatio-temporal properties. Personalized light exposure can be measured through wearable light loggers and dosimeters, including wrist-worn actimeters containing light sensors, yielding time series of an individual’s light exposure. There is growing interest in relating light exposure patterns to health outcomes, requiring analytic techniques to summarize light exposure properties. Building on the previously published Python-based *pyActigraphy* module, here we introduce the module *pyLight*. This module allows users to extract light exposure data recordings from a wide range of devices. It also includes software tools to clean and filter the data, and to compute common metrics for quantifying and visualizing light exposure data. For this tutorial, we demonstrate the use of pyLight in one example dataset with the following processing steps: (1) loading, accessing and visual inspection of a publicly available dataset, (2) truncation, masking, filtering and binarization of the dataset, (3) calculation of summary metrics, including time above threshold (TAT) and mean light timing above threshold (MLiT). The *pyLight* module paves the way for open-source, large-scale automated analyses of light-exposure data.

## 1 Introduction

Light exposure profoundly affects human physiology and behavior, including the entrainment of the circadian clock, the production of the hormone melatonin, alertness and mood (Blume et al. 2019; Vetter et al. 2022). These so-called “non-visual” effects of light are receiving attention from a variety of professionals, including neuroscientists, psychologists, lighting designers, architects, interdisciplinary scientists, regulators, and the general public (Houser and Esposito 2021; Stefani and Cajochen 2021; Vetter et al. 2022). Moreover, negative consequences from potentially insufficient indoor illumination during daytime and excessive light exposure in the evening from light-emitting devices, namely light in the short-wavelength range, are increasingly recognized. Recently, recommendations for optimal ambient light exposure levels have been developed, proposing optimal light levels for daytime, evening and nighttime light exposure (Brown et al. 2022) and exemplifying the translation of recent scientific findings in this field into practice (Spitschan and Joyce 2023).

In parallel to research uncovering the impact of light on human physiology and behavior, small, light loggers, which are wearable devices measuring personalized light exposure over extended periods, have been developed (Hartmeyer et al. 2022). Light loggers include commercial wrist-worn light sensors built into actimetry devices (e.g., Actigraph wGT3X-BT, Actigraph Headquarters; Pensacola, FL, USA; CamNTech Actiwatch 4/Motionwatch-8 CamNtech, Fenstanton, UK; Condor ActTrust1/2, Condor, São Paulo, Brazil), brooches and pendants (e.g., Lys, Lys Technologies, Copenhagen, Denmark, Condor ActLumus, Condor, São Paulo, Brazil) and research-grade near-corneal-plane light loggers (e.g. LuxBlick (Hubalek et al. 2006, 2010), lido (Stampfli et al. 2023)). There is a large and growing set of studies using light loggers (Aarts et al. 2017; Bierman et al. 2005; Campbell et al. 1988; Dumont and Beaulieu 2007; Figueiro et al. 2013; Hubalek et al. 2006, 2010; Jardim et al. 2011; Markvart et al. 2015; Okudaira et al. 1983; Savides et al. 1986; Scheuermaier et al. 2010; Smolders et al. 2013; Spitschan et al. 2022; Thorne et al. 2009; Wams et al. 2017; Woelders et al. 2017), differing in calibration properties, position, data pre-processing and recording intervals (Hartmeyer et al. 2022).

A dominant factor in light exposure is exposure to daylight (Münch et al. 2020), which is given by illumination due to the Earth’s rotation. However, an individual’s personal light exposure shows great variability over time (Webler 2019), depending on light availability in the environment (indoors vs. outdoors; different lighting and window designs, types of light sources used), activities (outdoor and indoor activities at work, school, at home) and individual behavior (eye movements). Due to this variability, light exposure data must be measured individually in a personalized fashion, and cannot be predicted simply from environmental measurements.

The availability of personalized light exposure data requires the development of analytical and statistical tools, for which a series of metrics have been proposed (Hartmeyer et al. 2022). While various open-source tools have been developed to perform analysis of light data in general, including the web-based *luox* platform (https://luox.app/, (Spitschan et al. 2021)), *LuxPy* (Smet 2020), and *colour* (Mansencal et al. 2022) for the calculation of quantities from spectral and colorimetric data, none of these calculate exposure metrics from time-series light exposure data.

To facilitate the analysis of light exposure data, we introduce an open-source Python module for the analysis of time-series data. This module, termed *pyLight*, is part of and extends the previously published open-source *pyActigraphy* Python package (Hammad et al. 2021), which implements functions for the loading, processing and analysis of actigraphy data. In this article, we will introduce the module, describe the contained functions and demonstrate the use of the module, with a specific focus on importing data, manipulating data, and calculating exposure metrics from the data. To our knowledge, *pyLight* is the only software package allowing for the convenient calculation of light metrics in a device-agnostic fashion.

## 2 Overview of pyLight

### 2.1 Overall architecture

The *pyLight* module has been designed to be used independently within the *pyActigraphy* package. The module interfaces with the existing infrastructure of *pyActigraphy*. The basis of *pyLight* is a generic class holding the light exposure data. Via multiple inheritance, this class provides access to a list of analysis metrics dedicated to light exposure data analysis. Derived classes can then easily be implemented to support and read various native file formats from different light exposure devices. An example of such a class is provided by the *GenLightDevice* class of *pyLight*.

### 2.2 Accessing data from different devices

In addition, for each device supported by *pyActigraphy* (e.g., Actigraph wGT3X-BT, Actigraph Headquarters; Pensacola, FL, USA; CamNTech Actiwatch 4/Motionwatch-8 CamNTech, Fenstanton, UK; Condor ActTrust1/2, Condor, São Paolo, Brazil), the *pyLight* base class is able to handle the light exposure data recorded by these specific devices through a common framework once exported in proprietary software to text-based files.

### 2.3 Calculation of light exposure metrics

*pyLight* implements a series of light exposure metrics. These exposure metrics can be applied to a range of different light logger-measured quantities, including photopic illuminance or melanopic equivalent daylight illuminance (mEDI), and other quantities derived from spectral or multi-channel light logger measurements. As the metrics are generally agnostic to the exact quantity, in the below, we use the placeholder term “light exposure intensity.”

#### 2.3.1 Aggregated statistics

Means, medians and other summary statistics can be calculated over the entire recording or over user-defined periods.

#### 2.3.2 Threshold-based metrics

*pyLight* allows for the calculation of the time spent above a user-defined threshold (time above threshold; TAT) as well as the mean light timing above threshold (MLiT) developed by Reid and colleagues (Reid et al. 2014). The MLiT metric is defined as:

$$MLiT(C)=\frac{\sum_{j=1}^{m}\sum_{k=1}^{n}j\times I_{jk}(C)}{\sum_{j=1}^{m}\sum_{k=1}^{n}I_{jk}(C)}$$

Where *I*_*jk*_ is 1 if the light exposure intensity is above the threshold *C* for the daily period j on the day k and 0 otherwise, *j* is the index of the daily period, m is the number of daily periods (e.g., 1440 for light exposure intensity data measured every minute) and n is the number of days in the recording. This variable computes the time of day around which the mean light exposure intensity above the threshold *C* is centered. The TAT is simply the number of minutes above the defined threshold *C. C* is user-defined, thereby allowing for the probing of several different thresholds.

#### 2.3.3 L5 and M10 metrics

Calculation of time of day and mean value of light exposure intensity during the 5 hours window of least exposure or the 10 hours of maximal exposure (van Someren et al. 1997). L5 and M10 metrics are extreme values within these periods, without regard to the average light exposure intensity.

#### 2.3.4 Inter-daily stability (IS) and intradaily variability (IV)

These metrics have primarily been used to quantify rest-activity rhythms obtained from actigraphy studies (Witting et al. 1990). IS quantifies the stability of the daily pattern across days, while IV represents its fragmentation across the day. IS and IV have recently been applied to light exposure patterns and related to rest-rhythms (Kim et al. 2020).

Mathematically, IS and IV are are defined as:

$$\begin{array}{c} IS=\frac{n\sum_{h=1}^{p}{\left({\bar{x}}_{h}-\bar{x}\right)}^{2}}{p\sum_{i=1}^{n}{\left(x_{i}-\bar{x}\right)}^{2}}\\ IV=\frac{n\sum_{i=1}^{n}{\left(x_{i+1}-x_{i}\right)}^{2}}{(n-1)\sum_{i=1}^{n}{\left(x_{i}-\bar{x}\right)}^{2}}, \end{array}$$

where ${\bar{x}}_{h}$ is the average light exposure intensity for period *h* across all days, *x*_*i*_ is the light exposure intensity during period *i*, $\bar{x}$ is the overall mean light exposure intensity, *n* is the number of periods contained in the recording and p is the number of daily periods. IS is simply the 24-h value of the chi-square periodogram, normalized by the number of periods, *n* (Sokolove and Bushell 1978).

#### 2.3.5 Transformations

Data can be transformed conveniently into logarithmic scales.

### 2.4 Masking and data selection

A crucial step prior to the analysis of the light exposure data is the processing of raw data, i.e., removing light data where the device was not worn, or covered by sleeves (for wrist worn devices). To implement these preprocessing steps, the *pyLight* module offers various methods to mask periods of data acquisitions, either by only reading a specific continuous period of data defined by a user-defined start and stop time, or by adding periods where the unwanted data are masked, thereby not contributing to any subsequent analysis. Such periods can either be defined manually or specified in a separate file for easier editing and storage (see online tutorial at https://ghammad.github.io/pyActigraphy/pyLight-DataManip.html#Masking). In addition, summary statistics, such as mean, median, percentiles or standard deviations, as well as MLiT or TAT, can be obtained for consecutive or arbitrary time windows.

At present, there is no consensus on how light exposure data should be processed. Users of *pyLight* must understand that inclusion and exclusion of specific periods based on criteria may lead to biases of data, and we recommend an in-depth analysis of selection criteria.

### 2.5 Resampling and filtering

Another critical aspect of data processing covered by *pyLight* is resampling and filtering data before analysis. Often, fluctuations occurring at the acquisition frequency (e.g. tenths of a second) are considered irrelevant for the analysis of light exposure levels. To deal with these fluctuations, the *pyLight* module offers the possibility to resample the data to a specified frequency and aggregate periods with a custom function (sum, mean, median, etc). The module also allows users to digitally filter specific frequencies using a Butterworth filter.

### 2.6 Thresholding and binarization

Sometimes it is useful to only consider data with values above a specific numerical threshold. Thresholding can either discard data below that threshold, or the data can be transformed into binary data, where data above the threshold are replaced with 1 and 0 otherwise. The *pyLight* module provides various methods to directly access raw, thresholded or binarized data.

For some metrics, (e.g. TAT and MLiT), it is possible to directly specify the threshold used for the computation of such metrics.

### 2.7 Documentation

The documentation of the *pyLight* module is integrated into the documentation of the *pyActigraphy* package, which contains installation and “Quick Start” instructions for users, information about the authors, a code of conduct and the code license. The documentation is generated automatically from source code annotations and published. The list of online tutorials for *pyActigraphy* has been extended with specific tutorials for the *pyLight* module. Such tutorials are particularly useful for teaching users with various levels of programming expertise how to use all the functionalities available in the module. They start with basic instructions about how to read and visualize light exposure data, progress with examples on how to manipulate light exposure data (masking, resampling, thresholding, etc) and finish with more advanced code lines to compute specific light exposure metrics.

### 2.8 Code availability

The code, documentation and are open-source and available on GitHub (https://github.com/ghammad/pyActigraphy). The code base repository is licensed under the GNU General Public License v3.0. When the module is used for calculations, please cite this paper along with the original pyActigraphy publication Hammad et al. (2021).

### 2.9 Contributing

Researchers wishing to contribute to the pyLight module are welcome to do so by issuing pull requests on the pyActigraphy GitHub repository (https://github.com/ghammad/pyActigraphy).

## 3 A worked example

### 3.1 Basics

The following worked example will guide the reader on how to use the *pyLight* module by performing the analysis of a publicly available data set of actigraphy data licensed under the CC0 1.0 Universal (CC0 1.0) Public Domain Dedication license (Angelova et al. 2020). The data, collected with the Respironics Actiwatch Spectrum Pro (Respironics, Bend, OR, USA), were analyzed in two other publications (Kusmakar et al. 2021; Mellor et al. 2019).

### 3.2 Requirements

To follow this example, basic knowledge and a working installation of Python is a prerequisite. Many operating systems (including macOS and Linux) already come with a Python installation, but for others, it needs to be installed. The reader is pointed to the official Python website (https://wiki.python.org/moin/BeginnersGuide/Download) for instructions, but this information can be conveniently obtained through any web search engine. In addition, the pyActigraphy package must be installed (see https://ghammad.github.io/pyActigraphy/index.html#installation for instructions). This can be done using *pip*, the Python package management system.

### 3.3 Example

As in any Python script, the first step consists in importing the necessary modules. As already mentioned, *pyLight* forms part of the pyActigraphy package. Therefore, we simply start by importing it. Other packages are used for convenience:


          
**import pyActigraphy**
# Import the logarithm base 10 function
from the base Python math package
**from math import log10**
# Package for tabular data manipulation
**import pandas as pd**
# Package for statistical analyses
**import pingouin as pg**
# Package for interactive plotting
**import plotly.graph_objects as go**

        

Now, we define the path to the data to analyze:


          
# Adapt the path to the actual path
fpath = ‘/path/to/data’

        

The data files are stored in two different directories. One, *CLIENT*, is for data from chronic insomnia participants and the other, *PARTNERS*, is for data from their respective partners.

#### 3.3.1 Individual data analysis

Since these data were acquired with Respironics devices, we use the dedicated reader function, *read_-raw_rpx*, from the IO module of pyActigraphy to read the input data file:


            
**raw = pyActigraphy.io.read_raw_rpx**(
  fpath+’CLIENT/
  C1025_Acti1_Treatmen-
  t_6_09_2016_2_52_00_PM_New_Analy-
  sis.csv’,
  language=‘ENG_UK’,
    period=‘6D’, # Restrict data to
  the first 6 days.
)

          

As usual in python, more information about this function can be obtained with:


            
help(pyActigraphy.io.read_raw_rpx)

          

The input file contains both locomotor activity and light data. The latter can easily be accessed through the attribute *light* which holds an object of class *LightRecording*:


            
raw.light

          

**Fig. 1:**
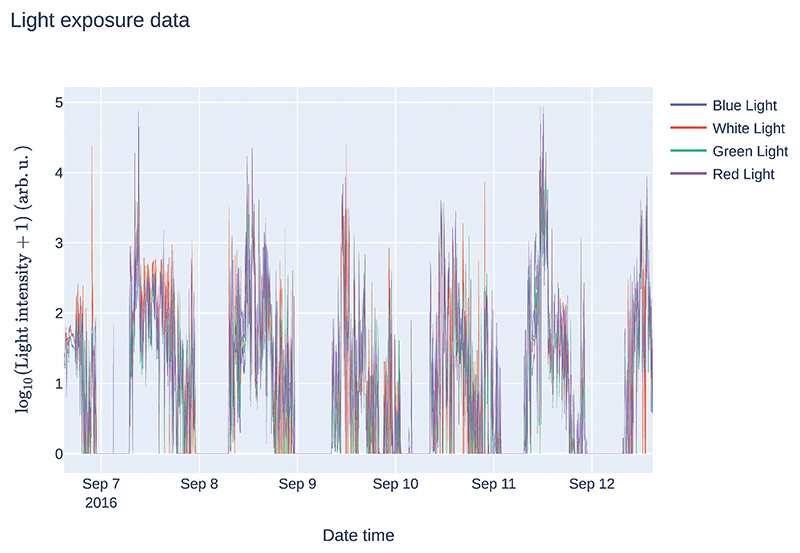
Time series of light exposure levels.

To inspect which light channels were found in this recording:


            
raw.light.get_channel_list()

          

returns a list with the names of the light channels. To visualize the data, we can plot all the light channels contained in the recording (Fig. 1):


            
layout = go.Layout(
  title=“Light exposure data”,
  xaxis=dict(title=“Date time”),
  yaxis=dict(title=“$\log_{10}
  (\mathrm{Light∼intensity}+1)$”),
  showlegend=True
)
fig = go.Figure(
  data=[
   go.Scatter(
    x=raw.light.get_channel(chan-
    nel).index.astype(str),
    y=raw.light.get_channel(chan-
    nel),
    name=channel,
   )
   for channel in raw.light.get_ch-
    annel_list()
  ],
  layout=layout
)
fig.show () # Interactive figure
    display

          

To save the figure to a file for later use, the function write_image can be used:


            
fig.write_image(‘fig_ind_light. png’, scale = 6)

          

Now that we have visually inspected our data, we can compute various light exposure metrics as follows:


            
# Exposure Levels with data thre-
sholded at *log*_10_(100 + 1).
light_expo_lvl = raw.light.light_ex-
posure_level(threshold=log10(100 + 
1))
light_expo_lvl.name = ‘Level_100’ #
Set the name for latter re-use
# Time above threshold (TAT), setting
the threshold at *log*_10_(100 + 1) and time in
minutes
light_tat = raw.light.TAT(thresh-
old=log10(100 + 1), oformat=“minute”)
light_tat.name = ‘TAT_100’
# Mean light timing (MLiT), setting the
threshold at *log*_10_(500 + 1)
light_mlit = raw.light.MLiT(thresh-
old=log10(500 + 1))
light_mlit.name = ‘MLit_500’

          

These function outputs are stored in pandas.Series objects that can easily be manipulated and concatenated into a summary table (*pandas.DataFrame*) that can be used for further statistical analysis or saved to a file:


            
results = pd.concat([light_expo_lvl,
light_tat, light_mlit],axis = 1)
results.to_csv(‘tab1.csv’)

          

#### 3.3.2 Group-level analysis

Visualization and inspection of individual data are an import step in any analysis. However, once this step is performed and the data are deemed suitable to analysis, it might be useful to read individual files by batch and compute metrics at the group level.

Here, we will show how to read all data files from both clients and partners, compute various metrics and compare the two groups.

To read multiple files at once, we can use the *read_-raw* function from the IO module of *pyActigraphy*:


            
**clients = pyActigraphy.io.read_raw**(
  fpath+’CLIENT/C×.csv’,
  reader_type=‘RPX’, period=‘6D’,
  language=‘ENG_UK’, n_jobs = 5
)
**partners = pyActigraphy.io.read_raw**(
  fpath+’PARTNER/P×.csv’,
  reader_type=‘RPX’, period=‘6D’,
  language=‘ENG_UK’, n_jobs = 5
)

          

Both the *clients* and *partners* objects store data, extracted from each individual files. Therefore, it is possible to loop over this individual data and compute the requested metrics.

Here, we will save the metric outputs to separate lists and then concatenate them to form a summary table:


            
# Declare empty lists
light_expo_lvls = []
light_tats = []
light_mlits = []

          


            
# Loop over all the LightRecording
objects contained in ‘clients’:
for iread in clients.readers:
  # Compute light exposure levels:
  light_expo_lvl = iread.light.
  light_exposure_level(threshold=-
  log10(100 + 1))
  light_expo_lvl.name = iread.name #
  set name to participant’s name
  light_expo_lvls.append(light_ex-
  po_lvl) # store

          


            
output in its dedicated list

# Compute time above threshold:
light_tat = iread.light.TAT(thresh-
old=log10(100 + 1), oformat=“time-
delta”)
light_tat.name = iread.name # set name
to participant’s name
light_tats.append(light_tat) # store
output in its dedicated list

# Compute mean light timing:
light_mlit = iread.light.MLiT
(threshold=log10(500 + 1))
light_mlit.name = iread.name # set
name to participant’s name
light_mlits.append(light_mlit) #
store output in its dedicated list

          

Once the loop is completed, individual results are concatenated into group-level summary tables:


            
clients_light_expo_lvl_results = pd.
concat(light_expo_lvls,axis = 1).T
clients_light_tat_results = pd.con-
cat(light_tats,axis = 1).T
clients_light_mlit_results = pd.con-
cat(light_mlits,axis = 1).T

          

Again, since the summary tables are *pandas. DataFrame*, it is possible to save them to csv files for further use:


            
clients_light_expo_lvl_results.
to_csv(‘tab_clients_expo_100.csv’)
clients_light_tat_results.to_csv
(‘tab_clients_tat_100.csv’)
clients_light_mlit_results.to_csv
(‘tab_clients_mlit_500.csv’)

          

To analyze the partner’s data, it simply requires to substitute the *clients* object by the *partners* object and then re-use the same code for iterating over individual files:


            
# Reset lists before storing indivi-
dual results:
light_expo_lvls = []
light_tats = []
light_mlits = []
for iread in partners.readers: # here,
clients has been changed to partners.
  light_expo_lvl = iread.light.
  light_exposure_level(threshold=-
  log10(100 + 1))
  light_expo_lvl.name = iread.name
  light_expo_lvls.append(light_ex-
  po_lvl)

  light_tat = iread.light.TAT
  (threshold=log10(100 + 1), ofor-
  mat=‘minute’)
  light_tat.name = iread.name
  light_tats.append(light_tat)
  
  light_mlit = iread.light.MLiT
  (threshold=log10(500 + 1))
  light_mlit.name = iread.name
  light_mlits.append(light_mlit)

  # Concatenate individual results
  for the partners
  partners_light_expo_lvl_results =
  pd.concat(light_expo_lvls,axis =
  1).T
  partners_light_tat_results = pd.
  concat(light_tats,axis = 1).T
  partners_light_mlit_results = pd.
  concat(light_
  mlits,axis = 1).T

  # Save results to.csv files
  partners_light_expo_lvl_results.
  to_csv(‘tab_part
  ners_expo_100.csv’)
  partners_light_tat_results.to_csv
  (‘tab_partner
  s_tat_100.csv’)
  partners_light_mlit_results.
  to_csv(‘tab_part
  ners_mlit_500.csv’)

          

**Fig. 2:**
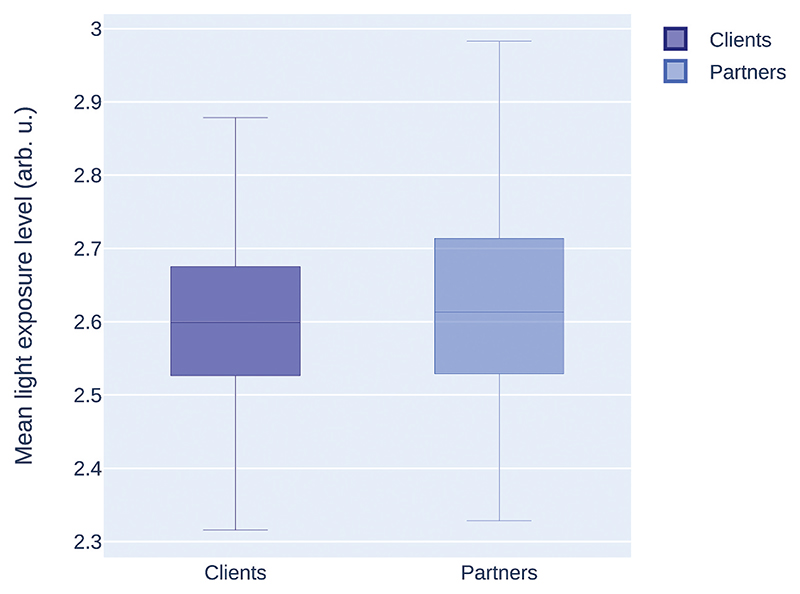
Boxplot of the mean light exposure level for clients and partners.

These summary files can now serve as input for statistical analyses, for example, or for visual representation of the results (Fig. 2):


            
fig = go.Figure()
fig.add _trace(go.Box(
  y=clients_light_expo_lvl_results.
  loc[:,’White Light’],
  name=‘Clients’,
  marker_color=‘darkblue,’
  #boxmean=True
))
fig.add _trace(go.Box(
  y=partners_light_expo_lvl_re-
  sults.loc[:,’White Light’],
  name=‘Partners’,
  marker_color=‘royalblue’,
  #boxmean=True
))
fig.update _layout
(yaxis_title=r’Mean light exposure
level,’ height = 500,width = 500);
# Display box plot
fig.show ()
# Save
fig.write _image(‘fig_grp_explevel.
png’, scale = 6)

          

## 4 Future directions

As more light loggers and dosimeters are being developed, the *pyLight* module will serve as a useful software entry point for the analysis of data produced by these devices. *pyLight* is able to perform its calculations in a device-agnostic manner, as long as the data stored on the device are accessible and use an open format that can be loaded into Python. Using a programmatic approach to light exposure facilitates sensitivity analyses in which thresholds in threshold-based metrics (such as TAT and MLiT) are varied systematically and the effect on a response variable is observed (Peeters et al. 2022). As the field of light logging and dosimetry matures, *pyLight* can be expanded to account for newly developed metrics, and can serve as a benchmark for alternative software solutions.

## 5 Conclusion

In conclusion, we presented *pyLight*, an extension to pyActigraphy, designed for the analysis of light exposure data. The module reports a series of light exposure metrics, which can be applied on a range of different existing file formats. We have presented a worked example demonstrating the use of the *pyLight* module on a previously published actigraphy data set containing light exposure.
